# Discussion on the Gradation and Interface Effects on the Dynamic Mechanical Behaviors of Hydraulic Concrete Based on Meso-Mechanical Simulation

**DOI:** 10.3390/ma18010015

**Published:** 2024-12-24

**Authors:** Chao Wang, Xinyu Zhou, Zhaopeng Deng, Xiaohua Wang, Sherong Zhang, Gaohui Wang, Peiyong Wei

**Affiliations:** 1 State Key Laboratory of Water Resources and Hydropower Engineering Science, Wuhan University, Wuhan 430072, China; wangchaosg@tju.edu.cn (C.W.); wanggaohui@whu.edu.cn (G.W.); 2 State Key Laboratory of Hydraulic Engineering Intelligent Construction and Operation & School of Civil Engineering, Tianjin University, Tianjin 300072, China; zhouxinyu_2002@163.com (X.Z.); tjudamdzp@163.com (Z.D.); tjuzsr@126.com (S.Z.); wepy@tju.edu.cn (P.W.)

**Keywords:** hydraulic concrete, aggregate gradation, construction-induced interface, mesoscale numerical simulation, dynamic mechanical behavior

## Abstract

Hydraulic concrete is quite different from normal concrete in the terms of aggregate gradation and construction-induced interfaces. To explore their influences on the dynamic mechanical behaviors of hydraulic concrete, several mesoscale numerical models with different aggregate gradations and interfaces were established and subjected to dynamic compressive or tensile loadings. The results show that aggregate gradation significantly affected hydraulic concrete failure patterns under dynamic loads, but interface effects were less obvious, and stressing uniformity improved with an increasing loading rate. The dynamic compressive and tensile strengths of hydraulic concrete showed a strain rate effect independent of gradation, but decreased with larger coarse aggregates, especially at higher rates. Weak-bonding interfaces significantly reduced strength at low loading rates, with a more pronounced effect on tensile strength than compressive strength. The results of this study provide a theoretical basis for the application of hydraulic concrete containing large-size aggregates in practical engineering.

## 1. Introduction

The aggregate in concrete usually takes up 70–80% by volume, which is considered as inert filler. It was recognized that the aggregate properties greatly affect the durability and mechanical performance of concrete material, including chemical and mineral composition, shape, roughness, weathering degree, hardness, and parent rock strength [1]. Moreover, the contributions from aggregates to concrete dynamic mechanical behaviors are considerable, especially at high loading rates. The existing of coarse aggregates can enhance the dynamic increase factors (DIFs) of specimens. As the maximum aggregate size increases, the material’s heterogeneity grows, resulting in a decrease in DIFs due to heightened dispersion. Adding more aggregates to concrete can significantly boost its dynamic strength when subjected to high strain rates [1,2,3]. Roller-compacted concrete (RCC) is distinct from normal concrete in terms of its mix design and construction methods [4]. RCC is characterized by a lower cement content and a very dry, hard mix that is compacted by mechanical vibration and rolling, as opposed to the workable consistency of normal concrete that requires internal vibration for compaction. RCC is known for its rapid construction speed, high strength, and cost-effectiveness, which are particularly advantageous in large-scale projects such as dams. The use of RCC can lead to significant time and cost benefits over conventional mass concrete dams, including higher placement rates, lower material costs, and reduced expenses associated with post-cooling and formwork. Additionally, RCC often incorporates a significant amount of fly ash as a partial replacement for Portland cement, which reduces the heat generated during hydration and thus the risk of thermal cracking, especially in large-volume constructions like dams. The distinct compaction process and layer structure of RCC contribute to its mechanical property differences compared to conventional concrete.

Recently, researchers have paid attention to the effect of concrete aggregate size, but the progress is relatively slow due to equipment size limitations that make experiments very difficult to conduct. Wang et al. [5] investigated the impact of coarse aggregate size on the thickness and micro-properties of the Interfacial Transition Zone (ITZ) and the mechanical properties of concrete, concluding that in concrete with a low water–cement ratio (0.3), the ITZ thickness increased with the size of coarse aggregate, leading to a decrease in concrete properties, while in concrete with a higher water–cement ratio (0.4), there was an optimal coarse aggregate size for the lowest ITZ thickness and highest strength. Albarwary et al. [6] conducted an experimental study that revealed the larger the particle size of coarse aggregate was, the weaker the ITZ was, leading to more unfavorable compressive strength in concrete. Mkpaidem et al. [7] investigated the influence of coarse aggregates with particle sizes ranging from 4.75 mm to 19.0 mm on concrete compressive strength and found that as the particle size of coarse aggregate increased, the compressive strength of concrete also increased, attributing this to the fact that smaller particle-sized coarse aggregates led to more capillary pores in hardened concrete, resulting in a looser ITZ pore structure. Therefore, the coarse aggregates with a maximum particle size less than 20 mm are commonly used in laboratory concrete tests to meet the uniformity requirement of specimens, ignoring the aggregate effect on the mechanical behaviors of concrete [8,9,10]. However, hydraulic concrete with a maximum aggregate size of 120 mm often possesses low porosity, a minimal interfacial transition zone (ITZ), and decreased water–cement ratio [11,12]. Therefore, the results of concrete mechanical tests in laboratory with small aggregate gradation cannot capture the mechanical behaviors of fully graded hydraulic concrete accounting for the true aggregate gradation effect.

The constitutive relation is a necessary basis for structural analysis, and the concrete strength criterion is the key in concrete constitutive model, which describes the peak strength under various loading conditions [13,14,15]. For hydraulic concrete with large aggregates, its failure criterion is not well understood, stressing the behaviors influenced by the special aggregate gradation and interface. The limited existing experimental investigations have verified that the lateral stress can enhance the strength of hydraulic concrete, yet an explicit model to forecast the behaviors of hydraulic concrete covering the aggregate gradation and interface effects under triaxial stress conditions has not been published [16,17]. In order to investigate the aggregate gradation effect of hydraulic concrete, mesoscopic numerical simulation provides a convenient and reasonable approach to study the failure mechanism and to establish the relations between meso-fracture and macro-mechanical behaviors of concrete-like materials [18,19,20]. The mechanical test based on mesoscopic numerical model can effectively avoid the problems of high laboratory test cost, equipment size limitation, and large measurement error. Collectively, they bring about complementary progress in dynamic strength criteria and predictive modeling techniques, thereby constructing a solid theoretical and computational foundation that is crucial for analyzing the dynamic mechanical behavior of hydraulic concrete [21,22].

In concrete meso-mechanics, the inner meso-structure of concrete can be generalized to be a multiphase composite structure, composed of coarse aggregate, mortar, and ITZ. To this end, Tian [23] established a two-dimensional mesoscale numerical model of hydraulic concrete with different aggregate gradations, including two-gradation (5~40 mm), three-gradation (5~80 mm), and four-gradation (5~150 mm), so as to take aggregate gradation effect into consideration, and the findings indicated that the aggregates gradation and particle size substantially influenced the compressive properties of concrete. Zhou et al. [24] utilized laser scanning and spatial cutting to create a dataset of real aggregate shapes and thoroughly examined the effects of aggregate shape and size on the uniaxial compressive strength of concrete. Moreover, to better understand the effects of aggregate size and proportioning on concrete’s static and dynamic properties, three-dimensional mesoscale simulations can be employed. Researchers have found that the aggregate content, particle size, and specimen dimensions primarily influence the split tensile strength of concrete under varying strain rates. Increased aggregate quantity and size at medium and low strain rates enhance strength; however, at high strain rates, the strength becomes less sensitive to changes in specimen size and aggregate characteristics [25]. Jin et al. [26] utilized mesoscopic simulation techniques to explore the damage patterns of concrete under two distinct conditions: varying aggregate particle sizes and specimen dimensions, at low strain rates. The study revealed that while the size of aggregate particles positively influenced the tensile strength of reinforced concrete, it began to limit compressive strength once a certain threshold is surpassed.

In sum, despite significant advancements in understanding the effects of aggregate gradation and size on concrete properties, there remains a gap in the literature concerning the specific influence of wide aggregate gradation and interface effects on fully graded hydraulic concrete. The existing research primarily focuses on specific gradations and strain rates, with limited exploration of the comprehensive dynamic behavior and mechanical properties across a broader range of conditions. To address these gaps, our study proposes a detailed investigation from a mesoscopic perspective, further exploring the aggregate and interfacial effects on wide-gradation hydraulic concrete. By leveraging mesoscale numerical models and conducting dynamic strength tests, we aim to provide a comprehensive understanding of how aggregate gradation and interfacial properties influence the dynamic mechanical behavior of fully graded hydraulic concrete.

This study aims to provide a deeper understanding of aggregate gradation and interface effects on fully graded hydraulic concrete. Initially, a mesoscale numerical model for hydraulic concrete materials was developed, and dynamic strength tests were conducted to validate the modeling approach, as well as the chosen constitutive model and parameters. Subsequently, mesoscale models for hydraulic concrete with four different aggregate gradations were established following this methodology. Dynamic compression and tension tests at different strain rates were conducted on concrete materials with various aggregate gradations to study their dynamic behavior and to refine the formula for strain rate effects [27]. Also, the study explored and discussed the impact of interface on the dynamic compressive and tensile strengths of hydraulic concrete. The findings of this study have substantially contributed to the knowledge of the dynamic mechanical properties of fully graded hydraulic concrete.

## 2. Materials and Methods

### 2.1. Simulation Method

Hydraulic concrete also consists of mortar matrix, coarse aggregates, and the ITZ. Considering the influence of mix design and layered structure on mechanical behaviors of hydraulic concrete, the static and dynamic behaviors of hydraulic concrete have been well explored by laboratory tests such as Split Hopkinson pressure bar (SHPB) tests, uniaxial, and triaxial compressive tests. The specimen production and laboratory test procedures have been explained in our previous studies in detail. Wang et al. [28] introduced the fabrication of concrete samples with a maximum aggregate size of 20 mm and the conduct of triaxial compressive tests to assess the mechanical properties of hydraulic concrete. However, it is noted that the aggregates used in specimen preparation were scaled to be 20 mm named as scaled hydraulic concrete in this study, so as to adapt the size of triaxial compressive equipment. In this case, the effects of aggregate gradation and interface cannot be well studied by traditional laboratory tests. Therefore, we used the meso-numerical simulation to obtain a deeper understanding of aggregate gradation and interface effects for hydraulic concrete.

In the numerical simulation, hydraulic concrete is simplified as homogeneous mortar, circular coarse aggregate following a certain gradation, ITZ with a fixed width. Since most coarse aggregates have an irregular shape, it is concluded that the aggregate shape has somewhat impacts on mechanical properties but will not change the basic mechanical rules of concrete [29]. The aggregate gradation and volume show more significant impacts on concrete mechanical properties than the aggregate shape does [28]. Thus, this article only used randomly generated circular element groups to simulate the aggregates in hydraulic concrete to improve computational efficiency [30,31,32,33,34,35,36]. There were four aggregate gradations set in this study, namely 5–20 mm, 5–40 mm, 5–60 mm, and 5–80 mm. The volume of coarse aggregate was set to be 50%, and the gradation distribution followed Equation (1) [37]. In practical engineering, the thickness of the ITZ in hydraulic concrete usually ranges between 20 and 50 μm, but many scholars simplify its thickness to between 0.5 and 2.0 mm in numerical simulation for computational efficiency [30,31,32,33,34,35,36]. Song et al. [38] found that increasing the thickness of ITZ from 0.5 mm to 2.0 mm would slightly affect the damage stage but not affect the macroscopic mechanical strength of concrete. Based on this, the ITZ thickness in this study was set to be 0.5 mm.

(1) $$P_{c}(d<d_{0})=P_{k}(1.065d_{0}^{0.5}d_{\max}^{-0.5}-0.053d_{0}^{4}d_{\max}^{-4}-0.012d_{0}^{6}d_{\max}^{-6}-0.0045d_{0}^{8}d_{\max}^{-8}-0.0025d_{0}^{10}d_{\max}^{-10}$$

where $P_{C}\left(d<d_{0}\right)\;$ denotes the volume fraction of aggregate within the cross-section having a particle size below $d_{0}$; $P_{k}$ denotes the aggregate volume fraction.

Different from normal concrete, hydraulic concrete exists in low-strength zones susceptible to shear loads, known as the interfaces, induced by the construction techniques of layered paving and rolling. In practice, the thickness of interfaces generally falls within the range from 0.5 cm to 2.0 cm [39,40]. Through a series of static and dynamic tests, it was found that hydraulic concrete containing 1.0 cm-thick interfaces could accurately characterize its macroscopic mechanical properties [10,41,42]. Thus, a thickness of 1.0 cm interface was also used to research the interface effect on dynamic mechanical properties of hydraulic concrete.

(2) $$v=\dot{\varepsilon }\times h$$

(3) $$v=\dot{\varepsilon }\times \frac{h}{2}$$

(4) $$v_{y}=\frac{2v}{b}y$$

where y=0 denotes the center position of the specimen.

**Figure 1:**
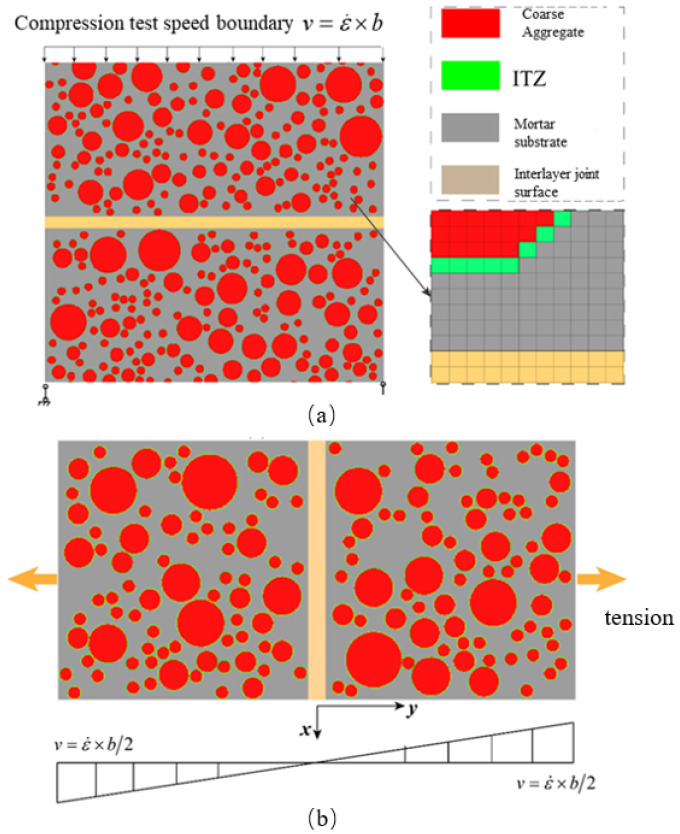
The mesoscopic simulation models of hydraulic concrete uniaxial compression and tension tests: (**a**) single-axis compression test; (**b**) uniaxial tensile test.

Figure 1 illustrates the mesoscopic numerical models of hydraulic concrete, established using the background grid projection method [43,44], for dynamic compressive and tensile experiments with the aspect ratios of 1 and 2, respectively. As shown in Figure 1, the hydraulic concrete was composed of coarse aggregate, mortar, ITZ, as well as the interface. The uniaxial dynamic compressive model in Figure 1a was constrained at the model base vertically, and a simultaneously constant velocity was applied on the top of the model to simulate the dynamic compressive loading. Similarly, the numerical model for dynamic tensile experiment as shown in Figure 1b was loaded as the same way, while a constant velocity was applied at both the top and bottom of the model to ensure a uniform stress distribution during the loading process. To ensure a constant loading rate, the loading velocity was quantified by Equations (2) and (3) for dynamic compressive and tensile experiments, respectively. And at the initial loading stage, the velocity boundary was increased gradually following Equation (4) in order to avoid the stress concentration.

### 2.2. Constitutive Model and Parameter Determination

#### 2.2.1. Concrete K&C Damage Model

**Figure 2:**
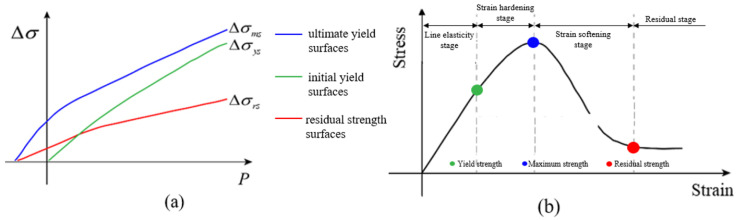
Schematic diagram of K&C constitutive strength surface: (**a**) schematic diagram of the strength surface in the K&C principal structure; (**b**) typical stress–strain relationship curve.

In the numerical simulation, an appropriate constitutive model for different material components is the prerequisite to accurately characterize the dynamic mechanical behaviors and failure modes of hydraulic concrete under various load conditions. Owing to the adeptness of K&C constitutive model, our study employed the K&C model for depicting the dynamic mechanical behaviors of these three mesoscopic components in concrete. The equations of the three strength surfaces are shown in Equations (5)–(7), and Figure 2 also illustrates the strength criteria of the K&C model.

(5) $$\Delta \sigma_{ys}=\left\{\begin{array}{cc} \;a_{0ys}+\frac{P}{\left(a_{1ys}+a_{2ys}\times P\right)} & P\geq \frac{f_{yc}}{3}\\ 1.35f_{t}+3P\times \left(1-\frac{1.35f_{t}}{f_{yc}}\right) & 0\leq P<\frac{f_{yc}}{3}\\ 1.35\times \left(P+f_{t}\right) & P<0 \end{array}\right.$$

(6) $$\Delta \sigma_{ms}=\left\{\begin{array}{cc} \;a_{0ms}+\frac{P}{\left(a_{1ms}+a_{2ms}P\right)} & P\geq \frac{f_{c}^{\prime }}{3}\\ \;\frac{1.5}{\Psi }\times \left(P+f_{t}\right) & 0\leq P\leq \frac{f_{c}^{\prime }}{3}\\ 3\times \left(\frac{P}{\eta }+f_{t}\right) & P<0,\lambda >\lambda_{m} \end{array}\right.$$

(7) $$\Delta \sigma_{rs}=a_{0rs}+\frac{P}{a_{1rs}+a_{2rs}P}$$

where $a_{xms}$, $a_{xys}$, and $a_{xrs}$ (for *x* = 0, 1, 2) are the parameters defining the strength surface, calibrated by triaxial tests; $P=(\sigma_{1}+\sigma_{2}+\sigma_{3})/3$ denotes the hydrostatic stress; ψ describes the meridional ratio reflecting the tensile-compressive behavior; $\;f_{yc}$, $\;f_{c}^{\prime }$, and $\;f_{t}$ denote the initial yield strength, the maximum compressive strength, and the ultimate tensile strength of the material, respectively; $\lambda_{m}$ represents the modified effective strain; λ stands for the damage variable, described as follows:

(8) $$\lambda =\left\{\begin{array}{cc} \int_{0}^{{\bar{\varepsilon }}_{p}}\;r_{f}^{-1}{\left[1+\frac{P}{r_{f}f_{t}}\right]}^{-b_{1}}d{\bar{\varepsilon }}_{p} & \;P\geq 0\\ \int_{0}^{{\bar{\varepsilon }}_{p}}\;r_{f}^{-1}{\left[1+\frac{P}{r_{f}f_{t}}\right]}^{-b_{2}}d{\bar{\varepsilon }}_{p} & \;P<0 \end{array}\right.$$

where ${\bar{\varepsilon }}_{p}$ represents the accumulated effective plastic strain; $d{\bar{\varepsilon }}_{p}$ denotes the incremental effective plastic strain at each step of integration; $r_{f}$ is the dynamic amplification factor for material strength, with $r_{f}=1$ corresponding to quasi-static loading conditions; $b_{1}$ and $b_{2}$ are parameters governing the rate of damage accumulation under compressive and tensile strains during the strain softening stage, respectively.

The bias stress at the damage surface is quantified by linear interpolation of Equation (9). In the scenarios of strain-hardening phase, the former is applied to describe the stress path from the initial yield surface $∆\sigma_{y}$ to the peak strength surface $∆\sigma_{m}$, while the latter is used to describe the strain-softening stage when the bias stress transitioning from $∆\sigma_{m}$ to $∆\sigma_{r}$.

(9) $$\Delta \sigma =\sqrt{3J_{2}}=\left\{\begin{array}{c} \eta (\Delta \sigma_{ms}-\Delta \sigma_{ys})+\Delta \sigma_{ms}\\ \eta (\Delta \sigma_{ms}-\Delta \sigma_{rs})+\Delta \sigma_{rs} \end{array}\right.$$

where $J_{2}=[(\sigma_{1}-\sigma_{2}{)}^{2}+(\sigma_{2}-\sigma_{3}{)}^{2}+(\sigma_{1}-\sigma_{3}{)}^{2}]/6$, represents the second stress invariant; $\sigma_{1}$, $\sigma_{2}$, and $\sigma_{3}$ denote the principal stresses along three orthogonal axes; η describes the strength surface scaling factor, which is adjusted based on the modified effective plastic strain; and $\Delta \sigma_{ys}$, $\Delta \sigma_{ms}$, and $\Delta \sigma_{rs}$ correspond to the initial yield, ultimate strength, and residual strength surfaces, respectively.

The association between η and λ is typically specified by the user. Conventionally, when ∆σ transiting from $∆\sigma_{y}$ to $∆\sigma_{m}$, η escalates from 0 to 1. Conversely, as ∆σ evolves from $∆\sigma_{m}$ to $∆\sigma_{r}$, η declines from 1 to 0. At the turning point where $\lambda =\lambda_{m}$, *η* is set to 1. The calculation formula for effective plastic strain considering volumetric damage is interpreted by Equation (10).

(10) $$\Delta \lambda =b_{3}f_{d}k_{d}\left(\varepsilon_{\nu }-\varepsilon_{\nu y}\right)$$

where $b_{3}$ denotes the regulatory parameter for the damage rate in materials during the strain-softening phase; $k_{d}$ acts as the intrinsic scaling multiplier; $\varepsilon_{v}$ and $\varepsilon_{vy}$, respectively, denote the volumetric and yield strains; $f_{d}\;$ is the constraint coefficient delineating the strength trajectory under triaxial tensile conditions, with its formal expression provided in Equation (11).

(11) $$f_{d}=\left\{\begin{array}{cc} 1-\left|\sqrt{3J_{2}}/P\right|/0.1 & 0<\left|\sqrt{3J_{2}}/P\right|<0.1\\ \;0 & \left|\sqrt{3J_{2}}/P\right|>0.1 \end{array}\right.$$

**Figure 3:**
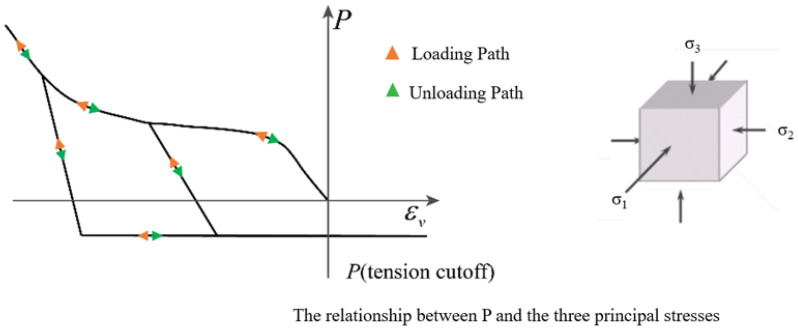
Schematic diagram of the equation of state in the K&C model.

In the K&C constitutive model, volumetric strain and hydrostatic pressure interaction is expressed by an equation of state, defined in LS-DYNA with the keyword *EOS_TABULATED_COMPACTION. Figure 3 illustrates the relationship between hydrostatic pressure and volume change, with the corresponding mathematical equation given in Equation (12).

(12) $$P=C\left(\begin{array}{c} \varepsilon_{\nu } \end{array}\right)+\gamma_{t}T\left(\begin{array}{c} \varepsilon_{\nu } \end{array}\right)E_{i}$$

In this context, function $C(\varepsilon_{v})$ consists of 10 sets of data $\left(\varepsilon_{v},P\right)$ input from outside the system; $\gamma_{t}$ denotes the ratio of specific heats; $T(\varepsilon_{v})$ is the thermodynamic parameter related to volumetric strain; and $E_{i}$ denotes the initial volumetric energy. It is important to note that, within the state equation under quasi-static loading conditions, the internal energy component can generally be disregarded.

#### 2.2.2. The Strain Rate Effect

As a dynamic constitutive model for common concrete materials, the K&C model takes the strain rate effect into account, which can affect the failure mode and strength of the material. In order to more accurately describe this change, strain rate enhancement parameter $\gamma_{f}$ is introduced to refine the ultimate strength surface, as illustrated by Equation (13).

(13) $$\Delta \sigma_{me}=\lambda_{f}\times \Delta \sigma_{m}\left(P/\gamma_{f}\right)$$

Hence, precise characterization of the strain rate effect on concrete materials will significantly enhance the simulation accuracy. To enhance the efficiency of the simulation, this study makes an appropriate simplification in calculating the strain rate effect of hydraulic concrete, using the same strain rate effect relations for mortar, interface and ITZ. Previous studies have shown that the existence of inertial effect overestimates the dynamic compressive strength of concrete [43].

(14) $$CDIF=\left\{\begin{array}{cc} \;0.0419\mathrm{lg}\dot{\varepsilon }+1.2165 & \dot{\varepsilon }\leq 30\;s^{-1}\\ 0.8988(\mathrm{lg}\dot{\varepsilon }{)}^{2}-2.8255\mathrm{lg}\dot{\varepsilon }+3.4907 & 30\;s^{-1}<\dot{\varepsilon }<1000\;s^{-1} \end{array}\right.$$

(15) $$TDIF=\left\{\begin{array}{cc} 0.26\mathrm{lg}\dot{\varepsilon }+2.06 & 10^{-4}\;s^{-1}\leq \dot{\varepsilon }\leq 1\;s^{-1}\\ 2.00\mathrm{lg}\dot{\varepsilon }+2.06 & 1\;s^{-1}<\dot{\varepsilon }<100\;s^{-1} \end{array}\right.$$

(16) $$CDIF=\left\{\begin{array}{cc} 0.0523\mathrm{lg}\dot{\varepsilon }+1.3138 & 10^{-6}\;s^{-1}\leq \dot{\varepsilon }<220\;s^{-1}\\ 2.6475{\left(\mathrm{lg}\dot{\varepsilon }\right)}^{2}-11.7664\mathrm{lg}\dot{\varepsilon }+14.4712 & 220\;s^{-1}\leq \dot{\varepsilon }\leq 1000\;s^{-1} \end{array}\right.$$

(17) $$\mathrm{TDIF}=\left\{\begin{array}{cc} 0.0598\mathrm{lg}\dot{\varepsilon }+1.3588 & 10^{-6}\;s^{-1}\leq \dot{\varepsilon }\leq 0.1\;s^{-1}\\ 0.5605(\mathrm{lg}\dot{\varepsilon }{)}^{2}+1.3871\mathrm{lg}\dot{\varepsilon }+2.1256 & 0.1\;s^{-1}\leq \dot{\varepsilon }\leq 50\;s^{-1} \end{array}\right.$$

**Figure 4:**
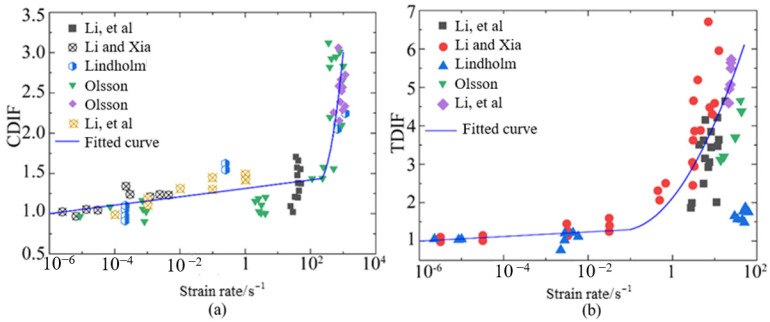
DIF relation of strain rate effect for coarse aggregate: (**a**) dynamic compressive strength; (**b**) dynamic tensile strength [46,47,48,49,50].

Thus, the relations of strain rate effect should eliminate the inertial effect and reveal the true material properties. In this study, the relations of strain rate effect for dynamic compressive and tensile strength, eliminating the inertial effect, are expressed by Equations (14) and (15), respectively [43,45]. With respect to strain rate effect for coarse aggregate, the relation was fitted by the data collected from the literature [46,47,48,49,50], as shown in Figure 4. Generally, the relation of strain rate effect for coarse aggregates can be described by Equations (16) and (17).

### 2.3. Verification of Simulation Model

The mesoscopic modeling method for hydraulic concrete proposed in this study has been proved by the authors’ previous works [10,51]. The ITZ and interface portions of the model are regarded as another mortar matrix with relatively lower strength [39,41,52]. It is reasonable to generalize ITZ with weakened mortar as it simplifies the complex modeling of ITZ in large-scale RCC arch dams, reduces computational costs significantly while approximating its role in overall stress distribution under various loads, thus facilitating efficient design and safety assessment [39]. Peng et al. [41] thought generalizing ITZ with weakened mortar was justified because it overcame the difficulties in precisely simulating the complex properties of ITZ. Using weakened mortar to generalize ITZ makes sense. In shear tests, ITZ is often a weak link, and weakened mortar, with its relatively low strength and shear resistance, can realistically mimic ITZ’s behavior under shear loads, predicting the shear failure mode more accurately. Meanwhile, it simplifies parameter settings and adjustments, making the simulation process more convenient and stable [52]. By adjusting the parameters of mortar to mimic ITZ’s mechanical characteristics, it strikes a balance between operability and accuracy of numerical simulation, reflecting the influence of ITZ on the meso-level mechanical properties of roller compacted concrete. Referring the experimental results in the literature [53], the K&C constitutive model parameters for the mortar matrix, ITZ, and aggregates were determined after iterative trial calculations, as shown in Table 1.

**Figure 5:**
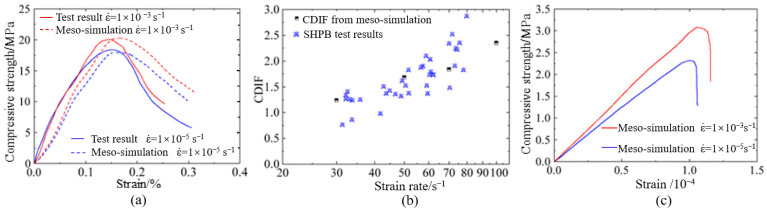
Validation of mesoscopic numerical model: (**a**) uniaxial compressive tests; (**b**) comparison of strain rate effect; (**c**) direct tensile tests.

The validation of the mesoscopic model and corresponding parameters is depicted in Figure 5. Figure 5a displays the results of uniaxial compressive tests on hydraulic concrete at the strain rates of 1 × 10^−5^ s^−1^ and 1 × 10^−3^ s^−1^ [54]. Although minor discrepancies were noted in the post-peak curves, the maximum peak strength error was merely 2.5%. To further investigate the applicability of the proposed mesoscopic numerical model under various loading conditions, the hydraulic concrete was subjected to uniaxial dynamic compressive tests at the strain rates of 30 s^−1^, 50 s^−1^, 70 s^−1^, and 100 s^−1^, and the computed CDIF values were compared with findings from the literature [53]. As illustrated in Figure 5b, the CDIF values obtained from numerical simulation closely matched those from SHPB test results. Moreover, in accordance with uniaxial tensile tests reported in the literature [55], mesoscopic numerical models for hydraulic concrete with three-gradation were established and subjected to uniaxial tensile tests at the strain rates of 1 × 10^−5^ s^−1^ and 1 × 10^−3^ s^−1^. Figure 5c shows that the tensile strength of the numerical model increased with the strain rate, matching the physical test results in the literature [55]. Therefore, the meso-model and simulation methods introduced in this paper are capable of accurately capturing the dynamic compressive and tensile behaviors of hydraulic concrete at high strains.

In traditional structural analysis, it is common to treat the concrete material as homogeneous, which effectively reduces the complexity of the constitutive model and enhances the computational efficiency. However, this treating method cannot fully describe the true dynamic mechanical properties of concrete. Thus, in this subsection, based on mesoscale models proposed herein, hydraulic concrete with four different aggregate gradations (5~20 mm, 5~40 mm, 5~60 mm, and 5~80 mm) are established for uniaxial compressive and tensile tests to get a deeper understanding of hydraulic concrete taking the aggregate gradation and interface into consideration.

## 3. Aggregate Gradation Effect on the Dynamic Strength of Hydraulic Concrete

### 3.1. Aggregate Gradation Effect on the Dynamic Compressive Strength

**Figure 6:**
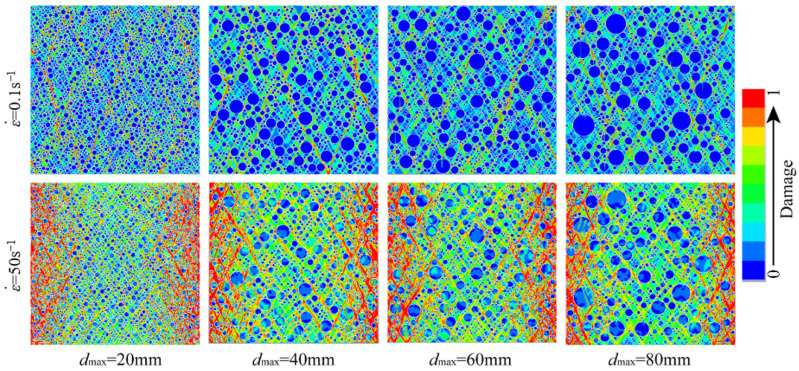
Compressive failure modes of hydraulic concrete with different aggregate gradations.

Figure 6 represents schematic illustrations of the compressive failure modes of hydraulic concrete specimens with various aggregate gradations subjected to different loading rates. As observed from Figure 6, the aggregate gradation did not change the general failure mode of the hydraulic concrete specimens, and crack development patterns remained broadly consistent at different strain rates. During the initial stages of loading, micro-cracks emerged within the mortar matrix and the ITZ area, and then these micro-cracks progressively evolved into diagonally penetrating fractures as the load continued.

It is noted that when the micro-cracks propagate and encounter the coarse aggregates, they extend around the aggregates via the weak ITZ regions at the relatively lower loading rates, while most coarse aggregates will bear a part of loads and remain in a stressed condition at the higher loading rates. Notably, the number of cracks and the extent of damage increase with higher strain rates. Thus, as the loading rate increases, the stressing uniformity among different material components becomes better. This phenomenon is attributed to the excessive loading rate, which retards the micro-cracks propagate along the weakest path and results in the more uniform stress distribution within the hydraulic concrete. On the other hand, the aggregate gradation also has a significant influence on the failure modes of the hydraulic concrete. It is obvious that the stress distribution of low gradation concrete is more uniform than that of high gradation no matter what the loading rate is. This further proves the retarding effect of larger coarse aggregate on the propagation of micro-cracks. Thus, the aggregate gradation will significantly affect the dynamic mechanical properties of hydraulic concrete. Many researchers have found that during the loading process, more uniform stress distribution and more severe material fracture occur in concrete-like materials at higher loading rates, which results in the higher strength enhancement [56,57,58].

In this study, the damage area ratio is introduced to quantitatively assess the material damage, which is defined as the ratio of damaged element area to total element area. The damage area ratio will significantly increase as the loading rate increases, while the damage area becomes relatively lower when the aggregate size becomes larger. For example, as shown in Figure 6, the damage area increased from 8.56% to 68.73% for the hydraulic concrete with the maximum aggregate size of 20 mm when the concrete was subjected from 0.1 s^−1^ to 50 s^−1^. However, when the maximum aggregate size increased from 20 mm to 80 mm under the loading rate of 50 s^−1^, the damage area decreased from 68.73% to 57.48%.

**Figure 7:**
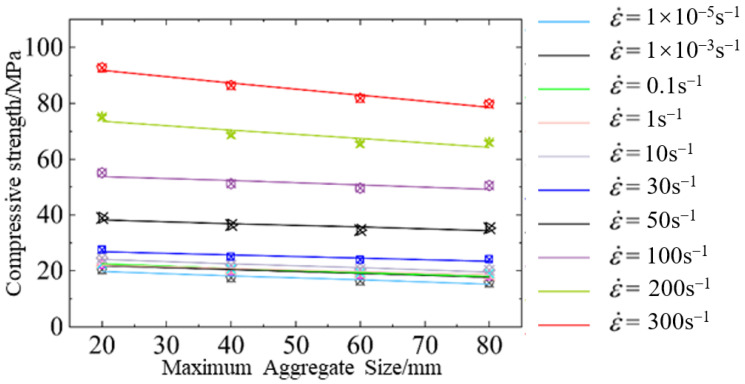
Aggregate gradation effect on the dynamic compressive strength of hydraulic concrete.

Figure 7 illustrates the relationship between maximum aggregate size and dynamic compressive strength of hydraulic concrete under various loading rates. As indicated in Figure 7, the loading rate and the maximum aggregate size are the primary factors influencing the dynamic compressive strength of hydraulic concrete. The dynamic compressive strength of hydraulic concrete progressively increased with the strain rate, and the increase in strength became significantly pronounced when the strain rate exceeded 30 s^−1^. Under an identical loading rate, the dynamic compressive strength of hydraulic concrete diminished as the aggregate size increased. This is because the presence of larger coarse aggregates restricted the propagation of micro-cracks and changed their propagation direction. Importantly, this retardation phenomenon of crack propagation induced by coarse aggregates became more distinct as the maximum aggregate size increased.

(18) $$CDIF=\left\{\begin{array}{cc} (A_{1c}+B_{1c}\times lg\dot{\varepsilon })\times {\left(\frac{d_{max}}{d_{0}}\right)}^{\delta_{1c}} & \dot{\varepsilon }\leq \dot{\varepsilon_{cr}}\\ (A_{2c}+B_{2c}\times lg\dot{\varepsilon }+C_{2c}\times (lg\dot{\varepsilon }{)}^{2})\times {\left(\frac{d_{max}}{d_{0}}\right)}^{\delta_{2c}} & \dot{\varepsilon }>\dot{\varepsilon_{cr}} \end{array}\right.$$

(19) $$CDIF=\left\{\begin{array}{cc} \left(1.16085+0.03481lg\dot{\varepsilon }\right)\times {\left(\frac{d_{max}}{d_{0}}\right)}^{0.04094} & \dot{\varepsilon }\leq 10\;s^{-1}\\ (2.75678-3.210051lg\dot{\varepsilon }+1.58079(lg\dot{\varepsilon }{)}^{2})\times {\left(\frac{d_{max}}{d_{0}}\right)}^{0.09291} & \dot{\varepsilon }>10\;s^{-1} \end{array}\right.$$

where $A_{1c}$, $A_{2c}$, $B_{1c}$, $B_{2c}$, $C_{2c}$, $\delta_{1c}$, and $\delta_{2c}$ are the fitting parameters; $d_{0}$ = 20 mm.

**Figure 8:**
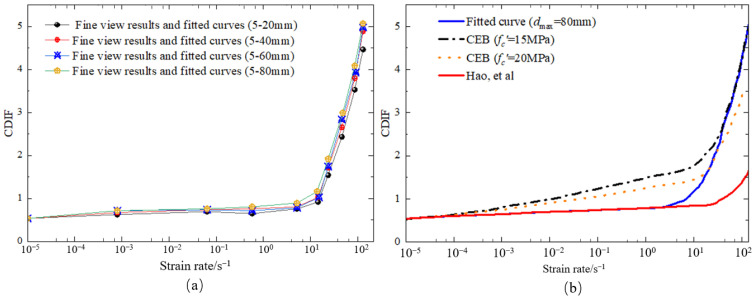
The coupled effect of aggregate gradation and strain rate on dynamic compressive strength: (**a**) strain rate effect for different aggregate gradations; (**b**) comparison with literature results [21].

It is obvious that hydraulic concrete with larger aggregates had a more significant strain rate effect at high loading rates than the results analyzed by Hao et al. [21] but matched with the CEB regulation better. It can be found that the aggregate gradation is considered by a power function in the strain rate effect for convenient engineering design of hydraulic concrete structures, which illustrates that the strain rate effect for hydraulic concrete with larger aggregates becomes more significant in strength enhancement under the dynamic loadings. Moreover, this strength enhancement induced by aggregate size is more distinct under a higher loading rate, and the critical strain rate is defined as $10\;s^{-1}$ under dynamic compression. The reason may be that when the loading rate exceeds the critical strain rate, the aggregate failure becomes more and more general as the loading rate increases. In this case, considering the aggregate gradation effect, Equation (18) is introduced herein to quantitatively assess the coupled effect of aggregate gradation and loading rate on dynamic compressive strength. Figure 8 depicts the correlation between strain rates and CDIF values for dynamic compressive strength of hydraulic concrete with various aggregate gradations. This correlation can be described by Equation (19) well after least squares fitting via the numerical results. 

### 3.2. Aggregate Gradation Effect on the Dynamic Tensile Strength

**Figure 9:**
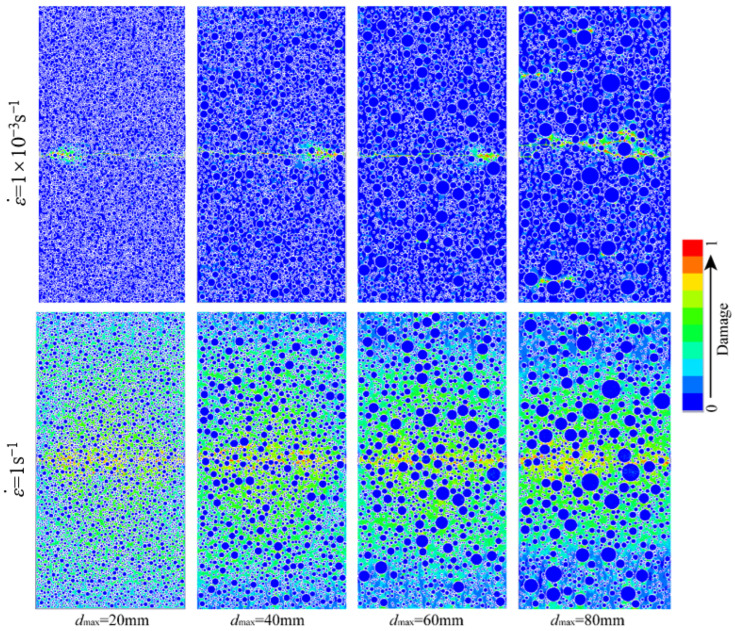
Tensile failure modes of hydraulic concrete with different aggregate gradations.

Figure 9 illustrates the tensile failure patterns of hydraulic concrete with varying aggregate gradations tested under different loading rates. As indicated by Figure 9, the strain rate and aggregate gradation significantly influenced the damage area and destruction extent in hydraulic concrete. At a relatively lower strain rate, micro-cracks primarily emerged in the central part of the concrete and developed into penetrating cracks along the mortar matrix or ITZs, which had poorer mechanical properties. When the loading rate increased, although the penetrating cracks still occurred at the center of the specimens, the tensile damage area enlarged significantly around the central fracture zone.

It is interesting that the central tensile damage area was influenced by the aggregate size, and the concrete with larger aggregates showed a smaller tensile damage area. However, this aggregate-affected damage phenomenon did not occur in the fracture patterns of hydraulic concrete subjected to low loading rates no matter what the aggregate gradation was. This may be explained by the tensile damage expansion of concrete, which can be effectively restricted by the large aggregates due to the pronounced strength difference between coarse aggregates and cement matrix. Thus, it is more likely to be fractured when the material damage is restricted in a limited area for hydraulic concrete with larger coarse aggregates, compared with the more uniformly damage distribution of hydraulic concrete with smaller coarse aggregates. Moreover, the change rule of damage area under dynamic tensile loads was similar to that under dynamic compressive loads. As illustrated in Figure 9, the damage area increased from 4.87% to 83.46% for the hydraulic concrete with the maximum aggregate size of 20 mm when the concrete was subjected from 10^−3^ s^−1^ to 1 s^−1^. However, when the maximum aggregate size increased from 20 mm to 80 mm under the loading rate of 1 s^−1^, the damage area decreased from 83.46% to 64.23%.

**Figure 10:**
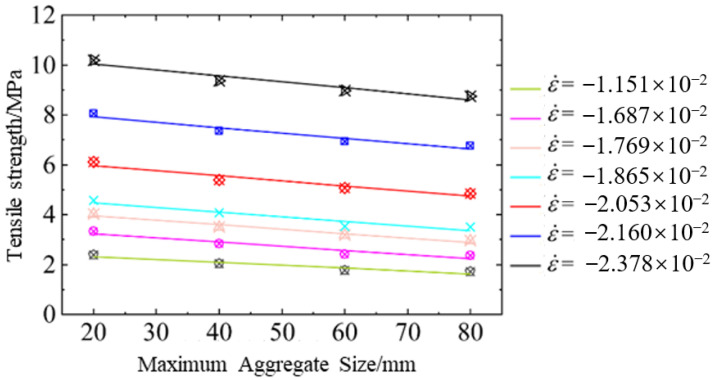
Aggregate gradation effect on the dynamic tensile strength of hydraulic concrete.

Figure 10 illustrates the relation between maximum aggregate size and dynamic tensile strength of hydraulic concrete subjected to different loading rates. The figure shows that the dynamic tensile strength of hydraulic concrete increased with the increasing strain rates, and this enhancement could be explained by a larger damage area being activated by the higher loading rate to bear and consume the energy of external loads. When the loading rate increased to a certain extent, the coarse aggregates could also be penetrated by the tensile cracks. Therefore, the enhancement of dynamic tensile strength could be constituted of larger damage area and coarse aggregate fracture. However, under the same loading conditions, the dynamic tensile strength progressively diminished with the enlargement of coarse aggregates, and this trend became particularly pronounced at higher loading rates. A rational reason for this observation lies in the fact that larger aggregates effectively acted as obstacles or barriers that interrupted the expansion of damage and the propagation of cracks. Consequently, as the size of the coarse aggregates increased, the area susceptible to tensile damage became more confined and limited, resulting in a smaller region where the external load or energy was consumed. Thus, the overall dynamic tensile strength diminished because the energy was dissipated over a smaller, more restricted tensile damage area that was constrained by the presence of larger coarse aggregates.

(20) $$\begin{array}{c} \mathrm{TDIF}=\left\{\begin{array}{ccccc} & \left(a_{1T}+b_{1T}\times \mathrm{lg}\dot{\varepsilon }\right){\left(\frac{d_{max}}{d_{0}}\right)}^{\gamma_{1T}} & & \dot{\varepsilon }\leq {\dot{\varepsilon }}_{cr} & \\ & \left(a_{2T}+b_{2T}\times \mathrm{lg}\dot{\varepsilon }+c_{2T}{\left(\mathrm{lg}\dot{\varepsilon }\right)}^{2}\right){\left(\frac{d_{max}}{d_{0}}\right)}^{\gamma_{2T}} & & \dot{\varepsilon }>{\dot{\varepsilon }}_{cr} & \end{array}\right. \end{array}$$

(21) $$\begin{array}{c} \mathrm{TDIF}=\left\{\begin{array}{ccccc} & \left(1.93024+0.19467\mathrm{lg}\dot{\varepsilon }\right){\left(\frac{d_{max}}{d_{0}}\right)}^{0.04007} & \dot{\varepsilon }\leq 1\;s^{-1} & & \\ & \left(1.82604-0.07062\mathrm{lg}\dot{\varepsilon }+0.63942{\left(\mathrm{lg}\dot{\varepsilon }\right)}^{2}\right){\left(\frac{d_{max}}{d_{0}}\right)}^{0.11918} & \dot{\varepsilon }>1\;s^{-1} & & \end{array}\right. \end{array}$$

where $a_{1T}$, $a_{2T}$, $b_{1T}$, $b_{2T}$, $c_{2T}$, $\gamma_{1T}$, and $\gamma_{2T}$ are the fitting parameters; $d_{0}$ = 20 mm.

**Figure 11:**
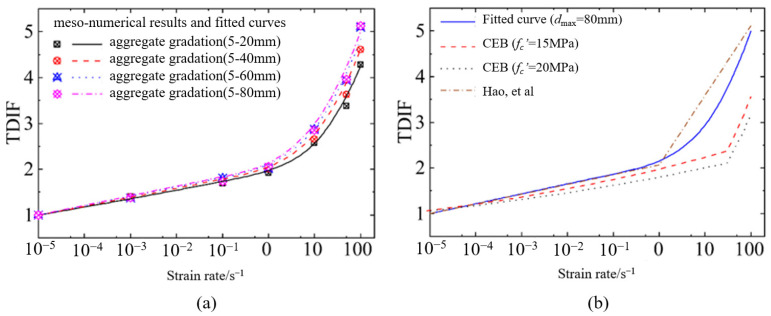
The coupled effect of aggregate gradation and strain rate on dynamic tensile strength: (**a**) strain rate effect for different aggregate gradations; (**b**) comparison with literature results [21].

Figure 11 depicts the correlation between strain rates and TDIF values for dynamic tensile strength of hydraulic concrete with various aggregate gradations. The graph indicates that the proposed tensile strain rate effect of hydraulic concrete obtained from the mesoscale simulation was more significant than that of CEB code, especially at high loading rates. In this condition, Equation (20) is introduced herein to consider the coupled effect of aggregate gradation and loading rate on dynamic tensile strength. This correlation can be described by Equation (21) well after least squares fitting via the numerical results. In Equation (21), the aggregate gradation is also considered by a power function in the strain rate effect for convenient engineering design of hydraulic concrete structures. Compared with Equation (19) under dynamic compressive loadings, the strength enhancement induced by aggregate size under dynamic tensile loadings was more distinct. The critical strain rate was defined as $1\;s^{-1}$ under dynamic tensile loadings, and when the loading rate exceeded the critical strain rate, the aggregate failure became more and more general as the loading rate increased.

## 4. Interface Effect on the Dynamic Strength of Hydraulic Concrete

Based on the mesoscopic modeling method proposed in this study, dynamic compressive and tensile testing models of hydraulic concrete with an interface of 1.0 cm were established as illustrated in Figure 1, in which the maximum aggregate size varied from 5 mm to 40 mm following the coarse aggregate grading curve in Equation (1). The mesoscopic numerical models were subjected to different loading rates so as to obtain their dynamic mechanical behaviors. Generally, the failure patterns of the mesoscopic numerical models having a weak-bonding interface were similar with those of models without interface. Thus, in this section, the interface effects on dynamic compressive and tensile strength of hydraulic concrete are emphasized and discussed in detail.

### 4.1. Interface Effect on the Dynamic Compressive Strength

**Figure 12:**
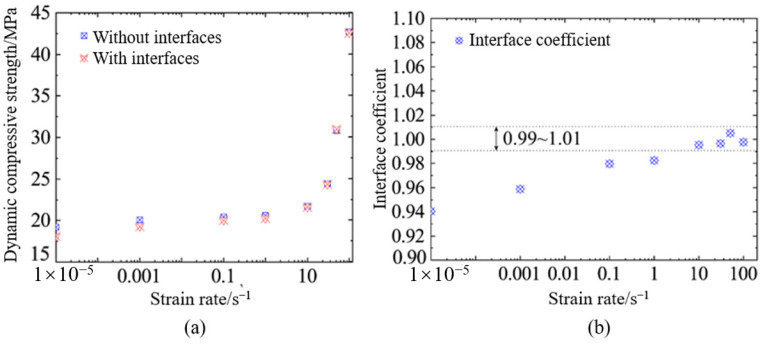
Interface effect of hydraulic concrete: (**a**) dynamic compressive strength; (**b**) interface coefficient for dynamic compressive strength.

Figure 12 illustrates the interface effect on the dynamic compressive strength of hydraulic concrete. It is observable that hydraulic concrete with interfaces usually had a relatively lower dynamic compressive strength compared to those without interfaces. However, this strength attenuation induced by weak-bonding interface was relatively obvious at a low loading rate and decreased with an increase in loading rate. As shown in Figure 12, the interface effect on dynamic compressive strength became negligible when the strain rate reached 10 s^−1^. This may be explained by the fact that the micro-cracks will not propagate along the weakest bonding path, and they will even propagate through the coarse aggregates under high loading rates.

(22) $$c=f_{d}^{w}/f_{d}^{wo}$$

where c means the interface coefficient of hydraulic concrete; $f_{d}^{w}$ and $f_{d}^{wo}$ represent the dynamic strength of hydraulic concrete with and without interface, respectively.

In order to further quantify the interface effect on dynamic compressive strength of hydraulic concrete, the interface coefficient is introduced herein, which can be described by Equation (22). Then, the interface coefficients of hydraulic concrete under various loading rates are described in Figure 12b. It was found that at quasi-static loading ($\dot{\varepsilon }$ = 1 × 10^−5^ s^−1^), the interface coefficient tended towards 0.94 and gradually approached 1 as the loading rate increased to or exceeded 10 s^−1^. This suggests that while the interface coefficients also showed a slight strain rate effect, the interface effect minimally impacted their dynamic compressive properties and could even be ignored at high loading rates. This was because the lower loading rates allowed for a uniform stress distribution within the specimen, leading to the initial failure of the weak bonding interface. However, at higher loading rates, hydraulic concrete rapidly fails, inhibiting stress propagating into the interface, which makes the interface effect on dynamic compressive strength of hydraulic concrete negligible at high loading rates.

### 4.2. Interface Effect on the Dynamic Tensile Strength

**Figure 13:**
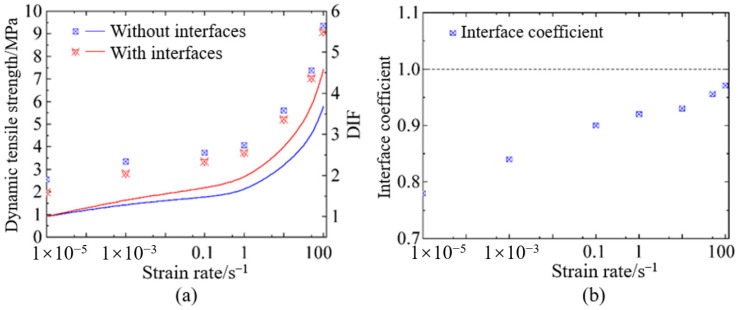
Interface effect of hydraulic concrete: (**a**) dynamic tensile strength; (**b**) interface coefficient for dynamic tensile strength.

With regard to the interface effect on dynamic tensile behaviors of hydraulic concrete, Figure 12 illustrates the interface effect on the dynamic tensile strength and the corresponding TDIF. It was observable that hydraulic concrete with interfaces usually had a relatively lower dynamic tensile strength compared to those without interfaces. However, this strength attenuation induced by weak-bonding interface was also obvious at a low loading rate and decreased with the increasing loading rate. Moreover, it was obvious that the interface effect on dynamic tensile strength was more significant than that on dynamic compressive strength. As shown in Figure 13a, the interfaces could still result in a tensile strength attenuation when the strain rate reaches 100 s^−1^, which was quite different from that of dynamic compressive strength.

Figure 13a also illustrate the fitted curves of strain rate effect for hydraulic concrete with and without interfaces, respectively. It is also interesting that although the concrete with an interface had a relatively lower dynamic tensile strength, its strain rate effect on tensile strength was more significant than that of concrete without an interface. When the interface coefficient as defined by Equation (22) was introduced to quantitively assess the interface effect, it is obvious in Figure 13b that the interface effect on dynamic tensile strength was also strain rate-sensitive. Under the quasi-static loading conditions ($\dot{\varepsilon }$ = 1 × 10^−5^ s^−1^), the interface coefficient had a value of 0.78 and increased to be greater than 0.94 when $\dot{\varepsilon }$ ≥ 1 s^−1^. The interface effect on the dynamic tensile strength of hydraulic concrete could gradually diminish at sufficiently high strain rates.

## 5. Conclusions

In this study, a mesoscale modeling method was proposed for dynamic compressive and tensile tests on hydraulic concrete. The reliability of the mesoscopic numerical model was validated against laboratory test results. Then, various mesoscale numerical models were established to explore the effects of aggregate gradation and weak-bonding interfaces induced by unique construction technique on dynamic mechanical behaviors. Some conclusions can be summarized as follows:

(1) The aggregate gradation usually had a significant influence on the failure patterns of hydraulic concrete under dynamic compressive and tensile loads, but the interface effect on the failure patterns was not obvious under dynamic loads. Moreover, as the loading rate increased, the stressing uniformity among different material components became better under both compressive and tensile loads. The stress distribution of low-gradation concrete was more uniform than that of high-gradation no matter what the loading rate was. This phenomenon was attributed to the excessive loading rate, which retarded the micro-cracks propagate along the weakest path and resulted in the more uniform stress distribution within the hydraulic concrete. Moreover, the damage expansion of concrete could be effectively restricted by the large aggregates due to the pronounced strength difference between coarse aggregates and cement matrix.
(2) The dynamic compressive and tensile strengths of hydraulic concrete demonstrated a pronounced strain rate effect, which was independent of aggregate gradation. However, under the same loading conditions, the dynamic compressive and tensile strength progressively diminished with the enlargement of coarse aggregates, with this trend being particularly pronounced at higher loading rates. A rational reason for this observation lies in the fact that larger aggregates acted as barriers that interrupted the damage expansion and the crack propagation. Furthermore, empirical formulae were proposed in this study to describe the strain rate effect on compressive and tensile strength of hydraulic concrete considering the unique aggregate gradation. Thus, the effect of aggregate gradation on the design parameters of hydraulic concrete structures under dynamic loading conditions could be generalized as an increase in the design parameters for engineering applications.
(3) The strength attenuation induced by weak-bonding interface was obvious at a low loading rate and decreased with the increasing loading rate. Moreover, it was obvious that the interface effect on dynamic tensile strength was more significant than that on dynamic compressive strength. When the interface coefficient was introduced to quantitatively assess the interface effect, it was obvious that the interface effect on dynamic compressive and tensile strength was strain rate-sensitive. However, the interface effect on the dynamic tensile strength of hydraulic concrete could gradually diminish at sufficiently high strain rates. Thus, in engineering applications, the interface effect on the design parameters of hydraulic concrete structures under dynamic loading conditions can be disregarded.

## Figures and Tables

**Table 1:** Parameters of the K&C constitutive model of mortar matrix, ITZ, and aggregates.

Basic Parameters				Strength Surface Parameters				Damage Parameters			
	Mortar	ITZ	Aggr.		Mortar	ITZ	Aggr.		Mortar	ITZ	Aggr.
ρ/kg·m^−3^	2000	1800	2650	$a_{0ys}$/MPa	5.58	5.58	7.70	$b_{1}$	1.60	1.60	0.75
v	0.18	0.20	0.25	$a_{1ys}$	0.625	0.625	0.513	$b_{2}$	1.35	1.35	3.21
G/GPa	10.63	8.65	20.65	$a_{2ys}$/×10^−9^ Pa^−1^	1.03	1.03	0.77	$b_{3}$	1.15	1.15	0.50
$f_{c}^{\prime }$/MPa	21	16	80	$a_{0ms}$/MPa	1.60	1.60	79	$I_{frac}$/mm	10	10	1.35
$f_{t}$/MPa	2.57	2.0	5.20	$a_{1ms}$	1.35	1.35	0.542	n	100	100	100
$d{\bar{\varepsilon }}_{p}$	0.5			$a_{2ms}$/×10^−9^ Pa^−1^	1.15	1.15	0.15	α	2.60	2.60	2.60
$\lambda_{t}$/×10^−3^	8.7	8.7		$a_{0rs}$/Pa	0	0	0	$\alpha_{c}$	0.283	0.283	0.283
ω			0.90	$a_{1rs}$	0.4417	0.4417	0.47	$\alpha_{d}$	1.74	1.74	1.74
				$a_{2rs}$/×10^−9^ Pa^−1^	4.732	4.732	0.20				

## Data Availability

The original contributions presented in this study are included in the article. Further inquiries can be directed to the corresponding author.

## References

[1] Rao M., Yang H., Lin Y., Li J., Shi Y. (2016). Influence of maximum aggregate sizes on the performance of RCC. Constr. Build. Mater..

[2] Hao Y.F., Hao H., Jiang G.P., Zhou Y. (2013). Experimental confirmation of some factors influencing dynamic concrete compressive strengths in high-speed impact tests. Cem. Concr. Res..

[3] Hao Y.F., Hao H. (2011). Numerical Evaluation of the Influence of Aggregates on Concrete Compressive Strength at High Strain Rate. Int. J. Prot. Struct..

[4] Wang C., Chen W., Hao H., Zhang S., Song R., Wang X. (2018). Experimental investigations of dynamic compressive properties of roller compacted concrete (RCC). Constr. Build. Mater..

[5] Wang Y., Zhang W., Wang J., Huang R., Lou G., Luo S. (2024). Effects of coarse aggregate size on thickness and micro-properties of ITZ and the mechanical properties of concrete. Cem. Concr. Compos..

[6] Albarwary I.H.M., Aldoski Z.N.S., Askar L.K. (2017). Effect of aggregate maximum size upon compressive strength of concrete. J. Duhok Univ..

[7] Mkpaidem N., Ambrose E., Olutoge F., Afangideh C. (2022). Effect of coarse aggregate size and gradation on workability and compressive strength of plain concrete. Appl. Sci. Environ. Manag..

[8] Wang X.-H., Zhang S.-R., Wang C., Liu F.-C., Song R., Wei P.-Y. (2018). Initial damage effect on dynamic compressive behaviors of roller compacted concrete (RCC) under impact loadings. Constr. Build. Mater..

[9] Wang X.-H., Zhang S.-R., Wang C., Song R., Shang C., Cao K.-L. (2018). Fragmentation-based dynamic size effect of layered roller compacted concrete (RCC) under impact loadings. Constr. Build. Mater..

[10] Zhang S.-R., Wang X.-H., Wang C., Song R., Huo H.-Y. (2017). Compressive behavior and constitutive model for roller compacted concrete under impact loading: Considering vertical stratification. Constr. Build. Mater..

[11] He Z., Deng H., Fan F., Tan J. (2018). Microstructure of four-graded roller compacted concrete. Constr. Build. Mater..

[12] Lin Y.Q., Shi Y., Guo D.M., Li J.Z., Yang H.Q. (2011). Study on Site Construction Technology of Four-Graded RCC. Adv. Mater. Res..

[13] Jiang J.-F., Xiao P.-C., Li B.-B. (2017). True-triaxial compressive behaviour of concrete under passive confinement. Constr. Build. Mater..

[14] Chen C., Zhang X., Hao H., Cui J. (2022). Discussion on the suitability of dynamic constitutive models for prediction of geopolymer concrete structural responses under blast and impact loading. Int. J. Impact Eng..

[15] Li B., Dai S., Zhan Y., Xu J., Guo X., Yang Y., Chen Y. (2022). Strength criterion of recycled aggregate concrete under triaxial Compression: Model calibration. Constr. Build. Mater..

[16] Zhang S., Wei P., Wang C., Wang G., Lu W., Cao K. (2021). Failure criteria calibration based on the triaxial compression behavior of roller compacted concrete (RCC). Mater. Struct..

[17] Deng R. (2002). Mechanical properties of roller compacted concrete. J. Southwest Jiaotong Univ..

[18] Gimenes M., Rodrigues E.A., Bitencourt L.A., Manzoli O.L. (2023). 2D mesoscale modeling of compressive fracture in concrete using a mesh fragmentation technique. Int. J. Solids Struct..

[19] Grassl P. (2023). 3D lattice meso-scale modelling of the effect of lateral compression on tensile fracture processes in concrete. Int. J. Solids Struct..

[20] Li H., Huang Y., Yang Z., Yu K., Li Q. (2022). 3D meso-scale fracture modelling of concrete with random aggregates using a phase-field regularized cohesive zone model. Int. J. Solids Struct..

[21] Hao Y., Hao H., Li Z. (2013). Influence of end friction confinement on impact tests of concrete material at high strain rate. Int. J. Impact Eng..

[22] Hao Y., Hao H., Li Z. (2010). Numerical analysis of lateral inertial confinement effects on impact test of concrete compressive material properties. Int. J. Prot. Struct..

[23] Tian R. (2008). Study on the Static, Dynamic Mechanical Properties of (Fully-Graded) Concrete Based on Mesomechanics. Master’s Thesis.

[24] Zhou Y., Jin H., Wang B. (2019). Modeling and mechanical influence of meso-scale concrete considering actual aggregate shapes. Constr. Build. Mater..

[25] Fang Q., Zhang J., Huang Y., Zhang Y. (2013). Investigation into three-dimensional mesoscale modelling of fully-graded concrete. Eng. Mech..

[26] Jin L., Yang W., Yu W., Du X. (2022). Influence of aggregate size on the dynamic tensile strength and size effect of concrete. J. Vib. Shock.

[27] Niu Y., Wang W., Su Y., Jia F., Long X. (2024). Plastic damage prediction of concrete under compression based on deep learning. Acta Mech..

[28] Wang C., Qiu Q., Wang X., Zhang S., Wang G., Wei P. (2024). Concrete Aggregate-Gradation Effect and Strength-Criterion Modification for Fully Graded Hydraulic Concrete. Materials.

[29] Jia J.-Y., Gu X.-L. (2021). Effects of coarse aggregate surface morphology on aggregate-mortar interface strength and mechanical properties of concrete. Constr. Build. Mater..

[30] Jin L., Yu W., Du X., Yang W.-X. (2020). Meso-scale simulations of size effect on concrete dynamic splitting tensile strength: Influence of aggregate content and maximum aggregate size. Eng. Fract. Mech..

[31] Du M., Jin L., Li D., DU X.L. (2017). Mesoscopic simulation study of the influence of aggregate size on mechanical properties and specimen size effect of concrete subjected to splitting tensile loading. Eng. Mech..

[32] Liu J.I.N., Wangxian Y.A.N.G., Wenxuan Y.U., Xiuli D.U. (2020). lnfluence of Maximum Aggregate Size on Dynamic Size Effect of Concrete Under Low Strain Rates: Meso-scale Simulations. Trans. Nanjing Univ. Aeronaut. Astronaut..

[33] Yu W.-X. (2019). Meso-Scale Simulation in Dynamic Size Effect on Compressive and Tensile Failure of Concrete Materials. Master’s Thesis.

[34] Jin L., Yu W., Du X., Yang W. (2019). Dynamic size effect of concrete under tension: A numerical study. Int. J. Impact Eng..

[35] Jin L., Yu W., Du X., Yang W.-X. (2019). Mesoscopic numerical simulation of dynamic size effect on the splitting-tensile strength of concrete. Eng. Fract. Mech..

[36] Liu J., Wenxuan Y., Xiuli D.U., Zhang S., Dong L.I. (2019). Meso-scale modelling of the size effect on dynamic compressive failure of concrete under different strain rates. Int. J. Impact Eng..

[37] Walraven J.C. (1981). Fundamental analysis of aggregate interlock. J. Struct. Div..

[38] Song Z., Lu Y. (2012). Mesoscopic analysis of concrete under excessively high strain rate compression and implications on interpretation of test data. Int. J. Impact Eng..

[39] Liu G., Hao J. (1996). Simplified numerical method in stress analysis of RCC arch dam with layered structure. J. Tsinghua Univ..

[40] Gu C.S., Song J.X., Fang H.T. (2006). Analysis Model on Gradual Change Principle of Effect Zones of Layer Face for RCCD. Appl. Math. Mech..

[41] Peng Y.J., Li B.K., Liu B. (2001). Numerical simulation of meso-level mechanical properties of roller compacted concrete. J. Hydraul. Eng..

[42] Qu Y., Peng Y., Li D. (2007). Numerical Simulation for Size Effect on Shear Strength of Roller Compacted Concrete Specimen. J. Water Resour. Archit. Eng..

[43] Naderi S., Zhang M. (2021). Meso-scale modelling of static and dynamic tensile fracture of concrete accounting for real-shape aggregates. Cem. Concr. Compos..

[44] Wu Y., Crawford J.E. (2015). Crawford, Numerical modeling of concrete using a partially associative plasticity model. J. Engineering Mech..

[45] Hao H., Zhou X. Concrete material model for high rate dynamic analysis. Proceedings of the 7th International Conference on Shock and Impact Loads on Structures.

[46] Li X., Lok T., Zhao J. (2005). Dynamic characteristics of granite subjected to intermediate loading rate. Rock Mech. Rock Eng..

[47] Li Y., Xia C. (2000). Time-dependent tests on intact rocks in uniaxial compression. Int. J. Rock Mech. Min. Sci..

[48] Lindholm U., Yeakley L., Nagy A. (1974). The dynamic strength and fracture properties of dresser basalt. Int. J. Rock Mech. Min. Sci. Geomech. Abstr..

[49] Olsson W. (1991). The compressive strength of tuff as a function of strain rate from 10-6 to 103/sec. Int. J. Rock Mech. Min. Sci. Geomech. Abstr..

[50] Li H.B., Zhao J., Li J.R., Liu Y.Q., Zhou Q.C. (2004). Experimental studies on the strength of different rock types under dynamic compression. Int. J. Rock Mech. Min. Sci..

[51] Wang X.H., Zhang S.R., Wang C., Song R., Shang C., Fang X. (2018). Experimental investigation of the size effect of layered roller compacted concrete (RCC) under high-strain-rate loading. Constr. Build. Mater..

[52] Peng Y., Li B., Qu Y. (2003). Numerical Simulation of Shear Test of Specimen Roller Compacted Concrete on Me-so-Level. J. Univ. Hydraul. Electr. Eng..

[53] Liu P. (2011). Experimental Research and Numerical Analysis on Dynamic Mechanical Properties of Concrete. Master’s Thesis.

[54] Lu D., Wang G., Du X., Wang Y. (2017). A nonlinear dynamic uniaxial strength criterion that considers the ultimate dynamic strength of concrete. Int. J. Impact Eng..

[55] Zhang K.-H., Wang H.-B., Tu J., Li C.-L., Zhong H. (2021). Dynamic tensile test of fully-graded roller compacted concrete. J. China Inst. Water Resour. Hydropower Res..

[56] Zhou X., Hao H. (2008). Modelling of compressive behaviour of concrete-like materials at high strain rate. Int. J. Solids Struct..

[57] Zhou R., Chen H.-M., Lu Y. (2020). Mesoscale modelling of concrete under high strain rate tension with a rate-dependent cohesive interface approach. Int. J. Impact Eng..

[58] Erzar B., Forquin P. (2014). Analysis and modelling of the cohesion strength of concrete at high strain-rates. Int. J. Solids Struct..

